# Supplementary material for: Prediction Models for Brain Distribution of Drugs Based on Biomimetic Chromatographic Data
Source: Molecules. 2022 Jun 7;27(12):3668. doi: 10.3390/molecules27123668 (PMC9227077; doi:10.3390/molecules27123668)
Supplement: Supplementary file 1 [file molecules-27-03668-s001.zip › Supplemetary Information_IAM_BBB_-special issue.pdf]

Supplementary Information for

Prediction models for brain distribution of drugs based on biomimetic chromatographic data

Theodosia Vallianatou, Fotios Tsopelas and Anna Tsantili –Kakoulidou

Contents

Supplementary Figures 3

Figure S1. DmodY bar plot for PLS model 4 of logKp,uu,brain including computational descriptors. 3

Figure S2. Permutation test (based on 100 permutations) for the PLS model 4 of $logK_{p,uu,brain}$. 3

Figure S3. Representative plot of observed vs predicted $logK_{p,uu,brain}$ values from external validation of PLS model 4. 4

Figure S4. Williams plots of standardized residuals versus the leverage 4

Figure S5. Representative plot of observed vs predicted logBB values from external validation of (**a**) Eq. (1); (**b**) Eq.2. 5

Figure S6. (a) DmodY bar plot for the PLS model 5 of logBB prediction including IAM retention factors; (b) DmodY bar plot for the PLS model 6 of logBB prediction including $logD_{7.4}$. 5

Figure S7. PLS models of logBB prediction including only the compounds with experimental $logk_{wAGP}$ values. 6

Figure S8. Permutation tests for the PLS models of logBB prediction. 6

Figure S9. Representative plot of observed vs predicted logBB values from external validation of PLS model 7

Figure S10. Williams plots of standardized residuals versus the leverage 7

Figure S11. Representative plot of observed vs predicted $logK_b$ values from external validation of (**a**) Eq. (7); (**b**) Eq. 11. 8

Figure S12. (a) Variable Importance ranking (b) Coefficient plot of the original variables in the PLS model 7. 8

Figure S13. (a) DmodY bar plot for the PLS model 8 of $logK_b$ prediction including IAM retention factors. (b) DmodY bar plot for the PLS model 9 of $logK_b$ prediction including logP and $logD_{7.4}$. 9

Figure S14. Permutation tests for the PLS models of $logK_b$ prediction 9

Figure S15. Representative plot of observed vs predicted $logK_b$ values from external validation of PLS model 10

Figure S16. Williams plots of standardized residuals versus the leverage 10

Figure S17. Representative plot of observed vs predicted $logV_{u,brain}$ values from external validation of (**a**) Eq. (15); (**b**) Eq.17 11

Figure S18. (a) DmodY bar plot for the PLS model 10 of $logV_{u,brain}$ prediction including IAM retention factors; (b) DmodY bar plot for the PLS model 11 of $logV_{u,brain}$ prediction including logP. 11

Figure S19. Permutation tests for the PLS models of $logV_{u,brain}$ prediction. 12

Figure S20. Representative plot of observed vs predicted $logV_{u,brain}$ values from external validation of PLS model 12

Figure S21. Plot of observed vs predicted $logV_{u,brain}$ values from PLS model 12. 13

Figure S22. DmodY bar plot for the PLS model 12 of $logV_{u,brain}$ 13

Supplementary Tables 14

Table S1. Compounds included in the data set; pharmaceutical classification, chromatographic data and lipophilicity parameters 14

Table S2. Brain disposition data, i.e., $K_{p,brain}$, $K_{p,uu,brain}$, $f_{u,brain}$, $V_{u,brain}$ for the investigated compounds 18

Table S3. Variable Importance to Projection (VIP) values in the PLS model 4 for $logK_{p,uu,brain}$ on the basis of biomimetic properties, lipophilicity and computational descriptors. 20

Table S4. Observed and predicted $logK_{p,uu,brain}$ values by the PLS model 4, based on computational descriptors 21

Table S5. Experimental and predicted logBB values by MLR and PLS models 22

Table S6. Experimental and predicted $logK_b$ values by MLR and PLS models 23

Table S7. Observed vs predicted $f_{u,brain}$ correlation: $f_{u,brain} = a*f_{u,brain,pred} + b$ 24

Table S8. Experimental and predicted $logV_{u,brain}$ values by MLR and PLS models 24

Supplementary Figures

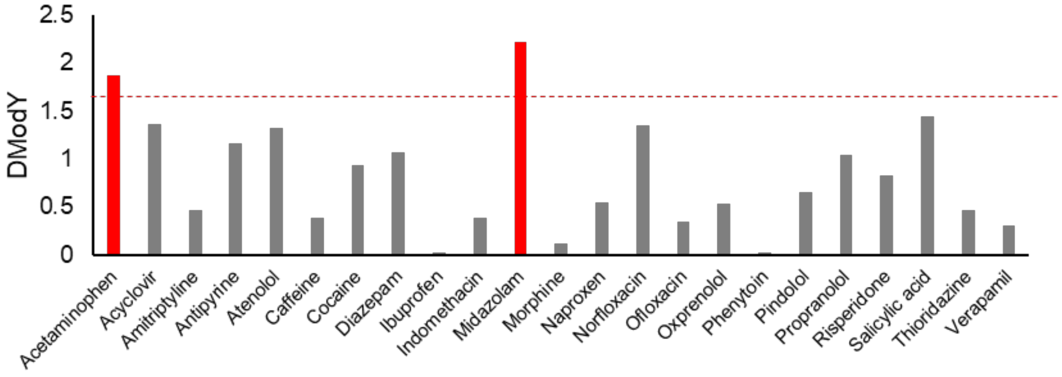

Figure S1. DmodY bar plot for PLS model 4 of logKp,uu,brain including computational descriptors. The red dashed line indicates the critical limit (1.65). Acetaminophen (1) and midazolam (11) extending it are highlighted with red bars.

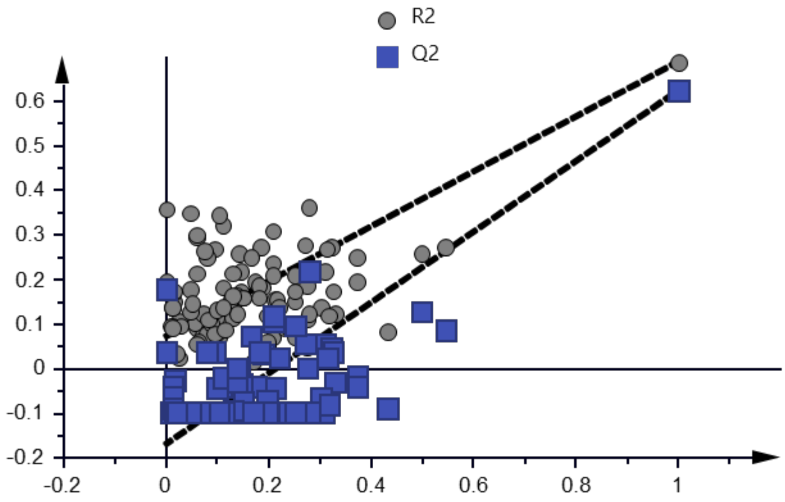

Figure S2. Permutation test (based on 100 permutations) for the PLS model 4 of $\log K_{p,uu,brain}$.

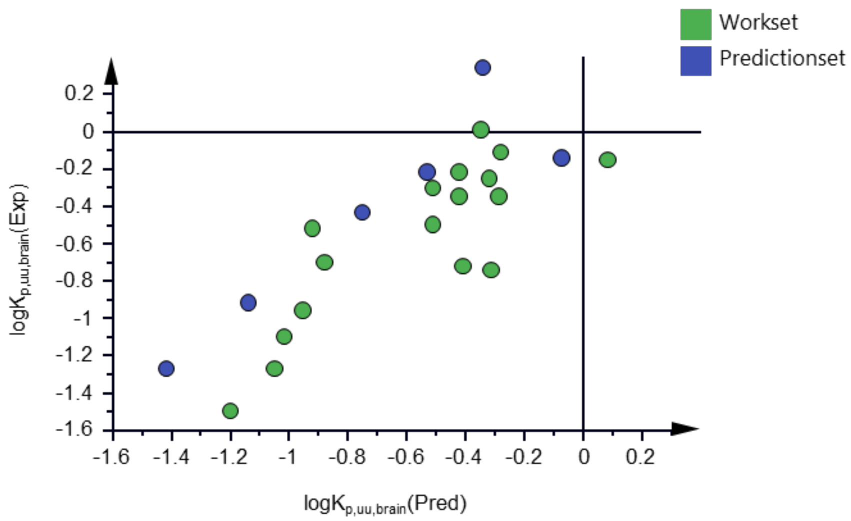

Figure S3. Representative plot of observed vs predicted log$K_{p,uu,brain}$ values from external validation of PLS model 4.

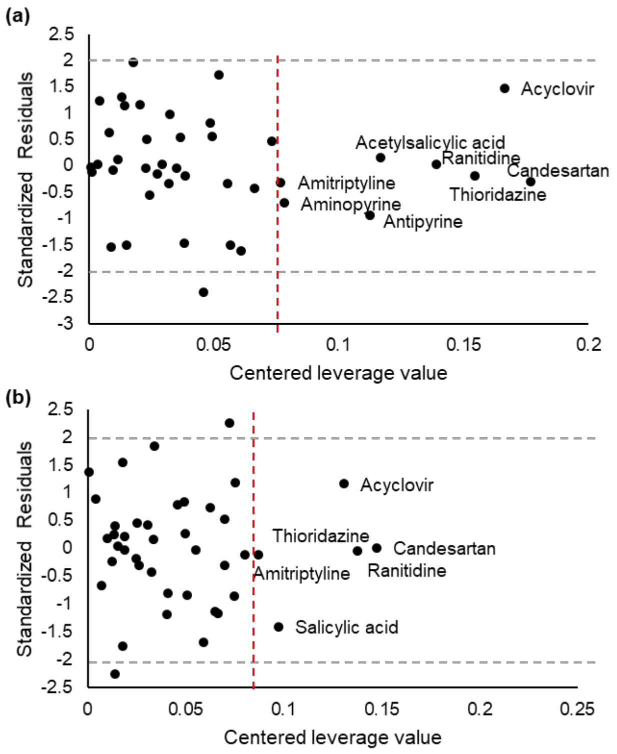

Figure S4. Williams plots of standardized residuals versus the leverage; (a) calculated from Eq.1; **(b)** calculated from Eq.2. Dashed grey lines indicate the ± 2*standard deviation space of the standardized residuals. Dashed red line indicates the h* value of each data set.

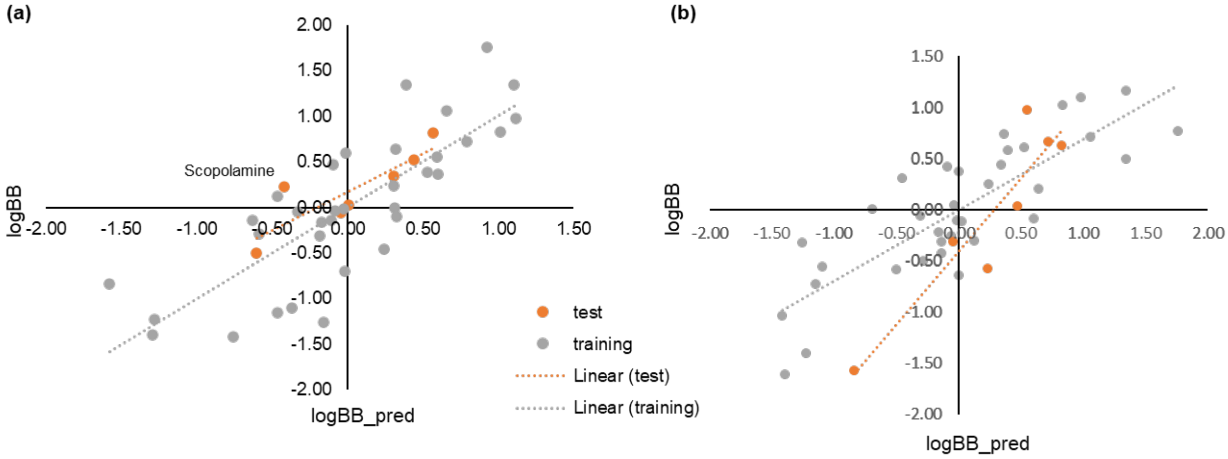

Figure S5. Representative plot of observed vs predicted logBB values from external validation of (**a**) Eq. (1); (**b**) Eq.2.

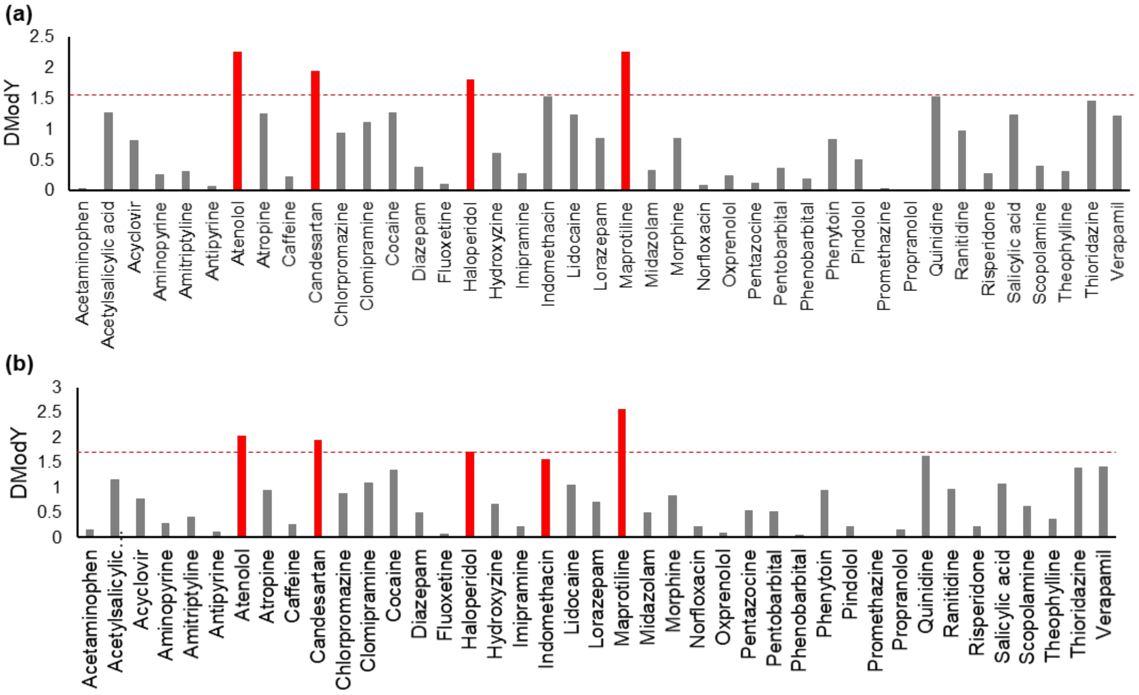

Figure S6. (a) DmodY bar plot for the PLS model 5 of logBB prediction including IAM retention factors; (b) DmodY bar plot for the PLS model 6 of logBB prediction including $\mathbf{logD_{7.4}}$**.** The red dashed line indicates the critical limit and the compounds extending it are highlighted with red bars.

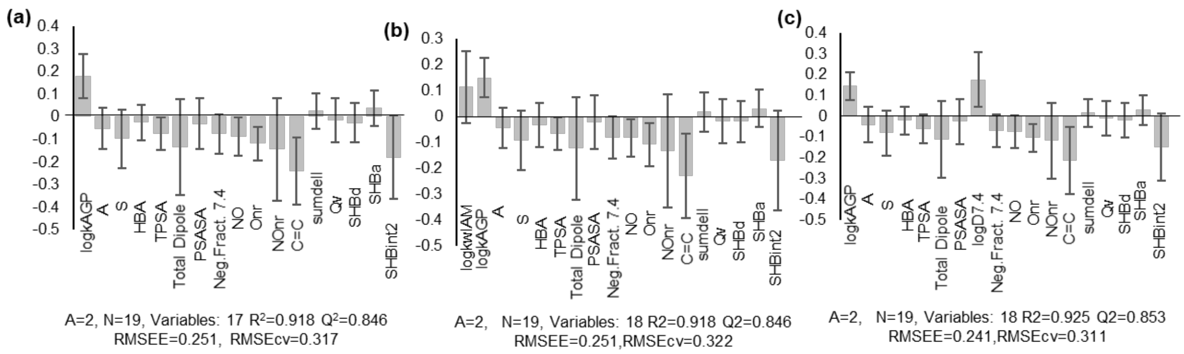

Figure S7. PLS models of logBB prediction including only the compounds with experimental logk$_{wAGP}$ values. (a) PLS model based only on logk$_{wAGP}$ and computational descriptors; **(b)** PLS model based on logk$_{wAGP}$, logk$_{wIAM}$ and computational descriptors; **(c)** PLS model based on logk$_{wAGP}$, logD$_{7.4}$ and computational descriptors.

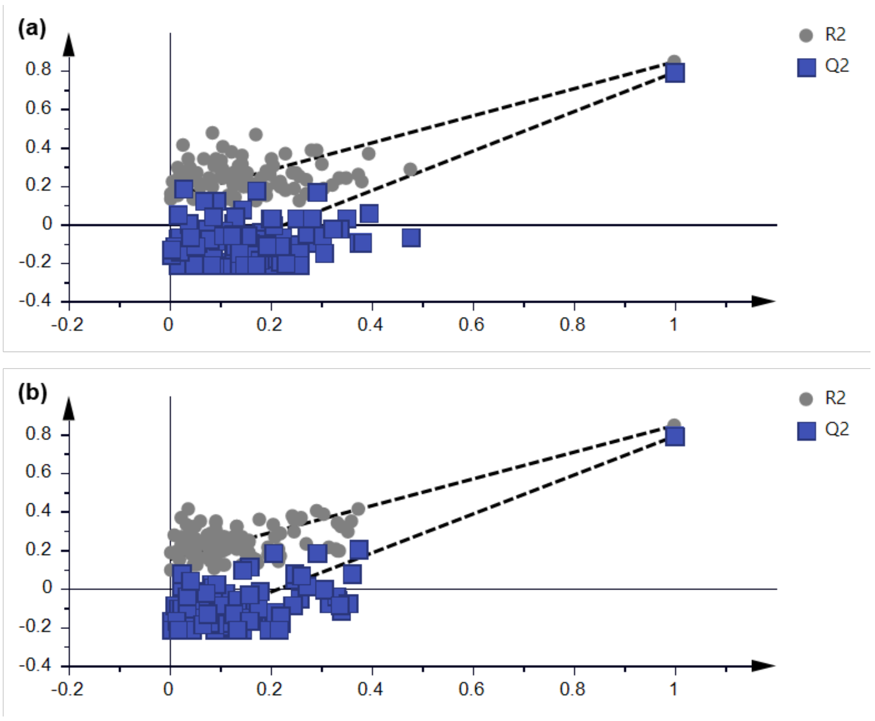

Figure S8. Permutation tests for the PLS models of logBB prediction. (a) Permutation test (100 permutations) for the PLS model 5 including IAM retention factors; **(b)** Permutation test (100 permutations) for the PLS model 6 including logD$_{7.4}$.

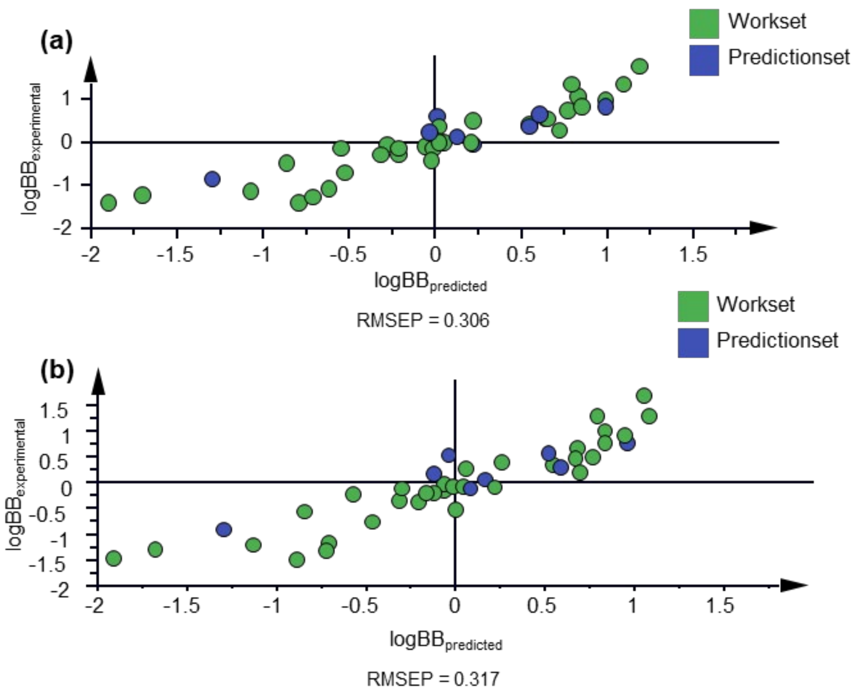

Figure S9. Representative plot of observed vs predicted logBB values from external validation of PLS model including (a) IAM retention factors/PLS model 5; **(b)** lipophilicity parameters/PLS model 6.

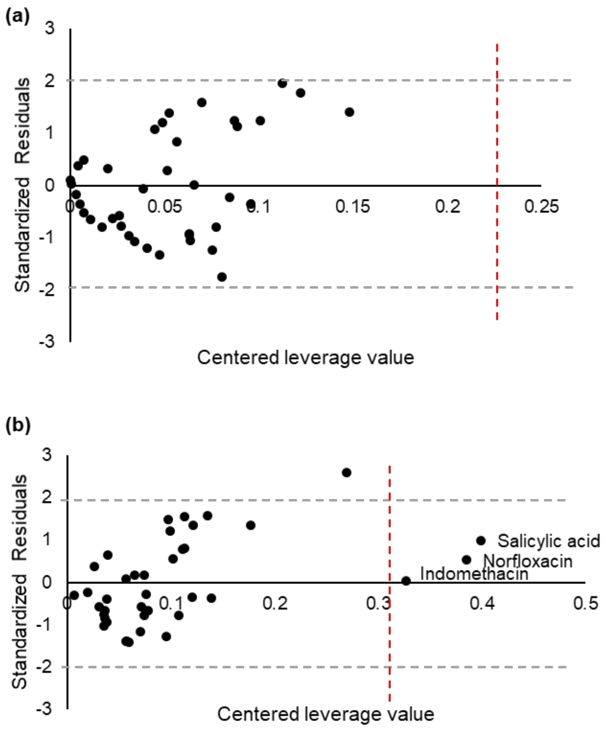

Figure S10. Williams plots of standardized residuals versus the leverage; (a) calculated from Eq.7; **(b)** calculated from Eq.11. Dashed grey lines indicate the ± 2*standard deviation space of the standardized residuals. Dashed red line indicates the h* value of each data set.

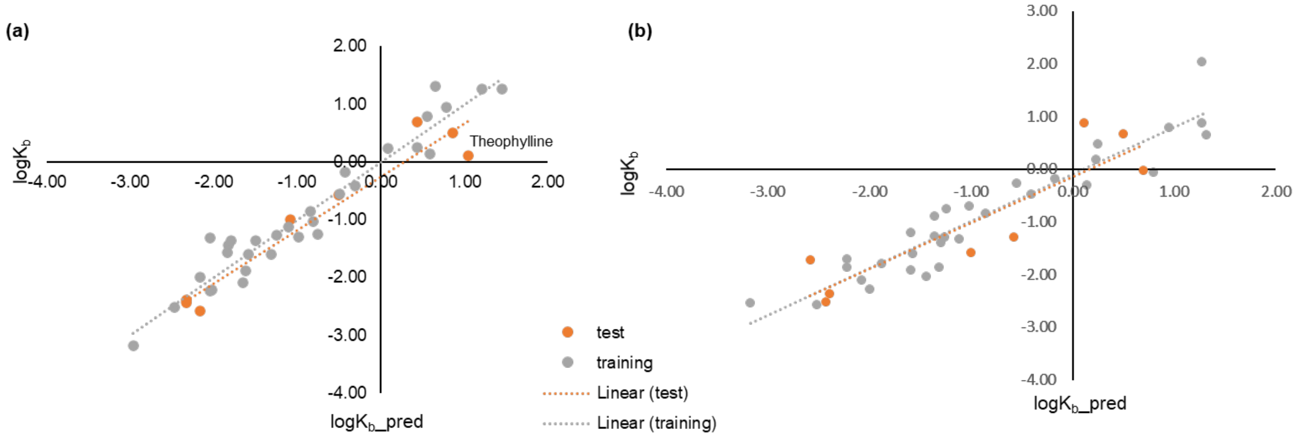

Figure S11. Representative plot of observed vs predicted logK$_b$ values from external validation of (**a**) Eq. (7); (**b**) Eq. 11.

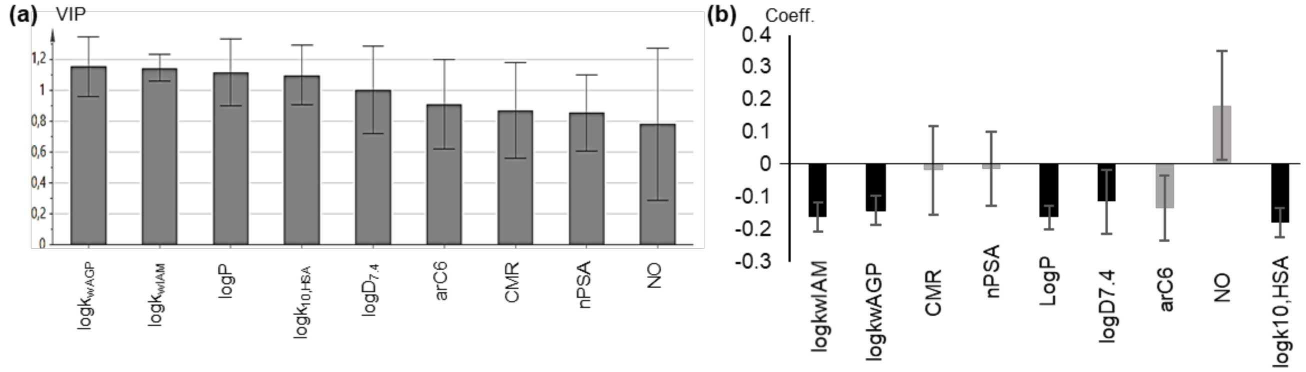

Figure S12. (a) Variable Importance ranking (b) Coefficient plot of the original variables in the PLS model 7. Original variables with VIP>1 are highlighted in black.

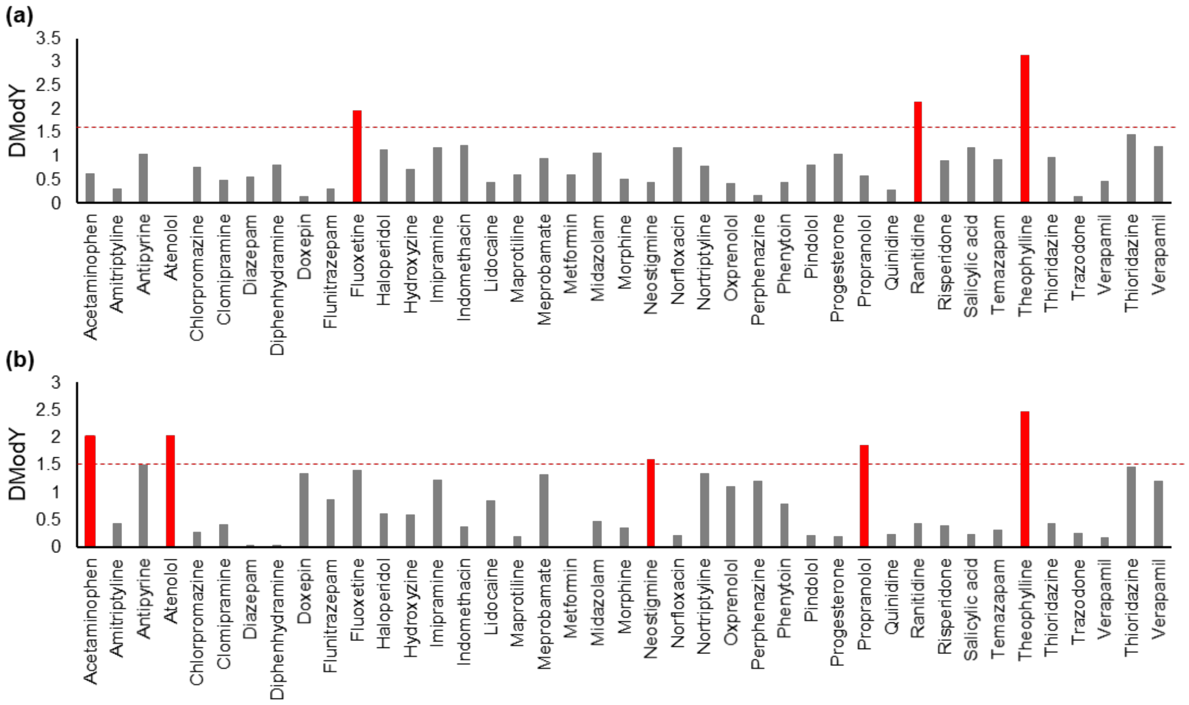

Figure S13. (a) DmodY bar plot for the PLS model 8 of logK$_b$ prediction including IAM retention factors. (b) DmodY bar plot for the PLS model 9 of logK$_b$ prediction including logP and logD$_{7.4}$. The red dashed line indicates the critical limit and the compounds extending it are highlighted with red bars.

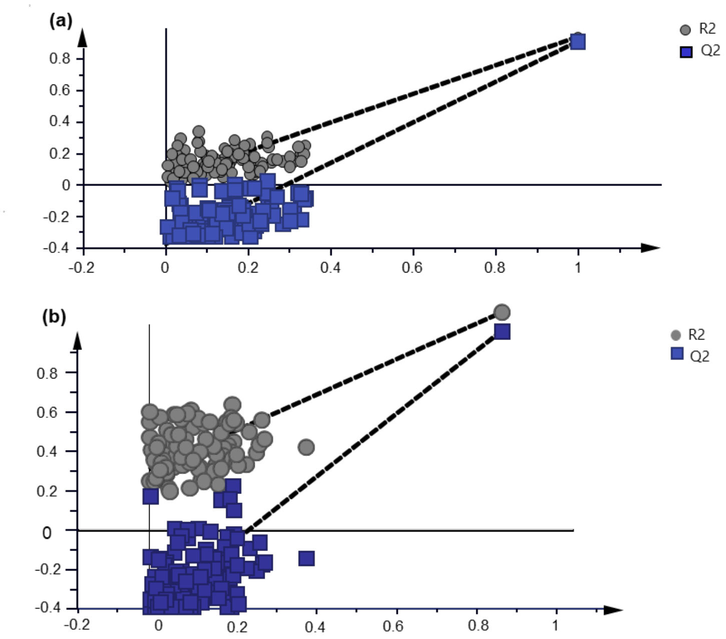

Figure S14. Permutation tests for the PLS models of logK$_b$ prediction. (**a**) Permutation test (100 permutations) for the PLS model including IAM retention factors; (**b**) Permutation test (100 permutations) for the PLS model including logP and logD$_{7.4}$.

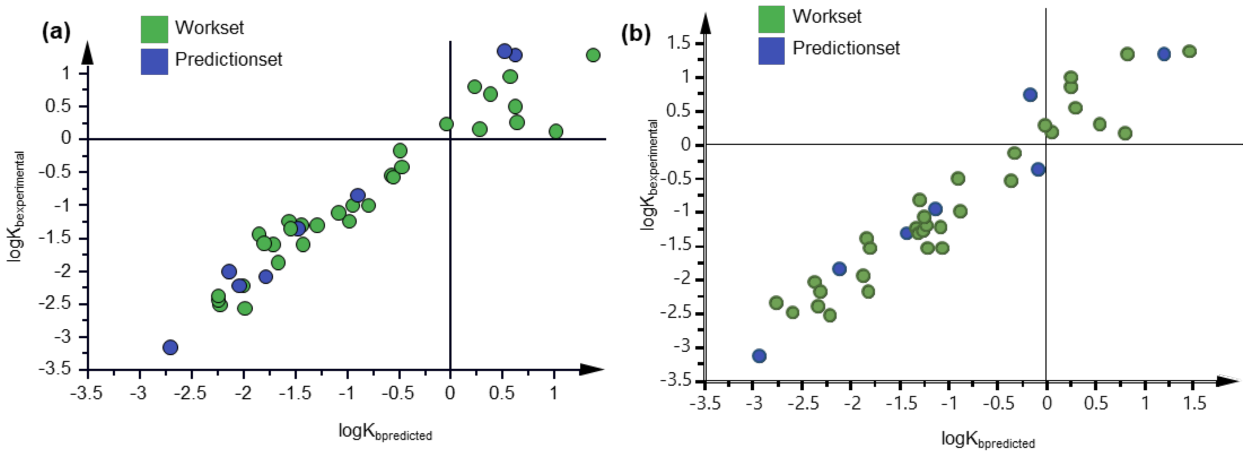

Figure S15. Representative plot of observed vs predicted logK_b values from external validation of PLS model including (a) IAM retention factor- PLS model 8; **(b)** lipophilicity parameters- PLS model 9.

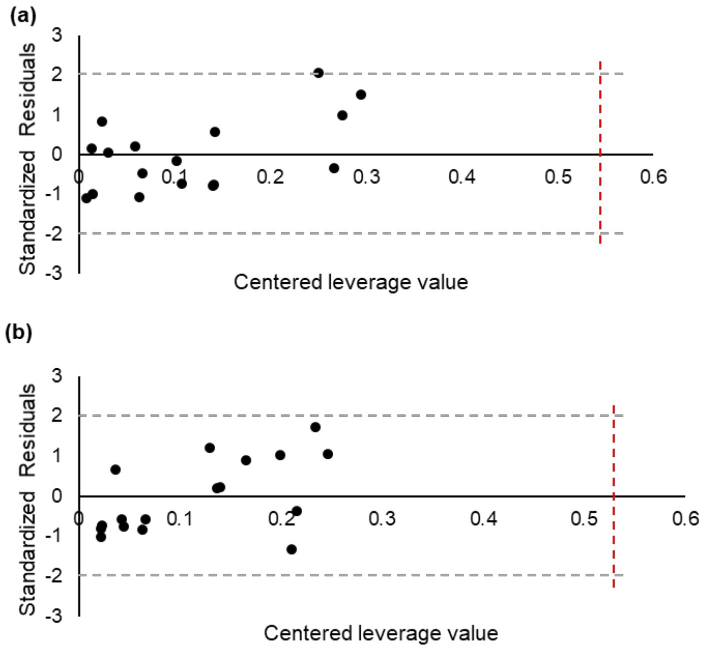

Figure S16. Williams plots of standardized residuals versus the leverage; (a) calculated from Eq.15; **(b)** calculated from Eq.17. Dashed grey lines indicate the ± 2*standard deviation space of the standardized residuals. Dashed red line indicates the h* value of each data set.

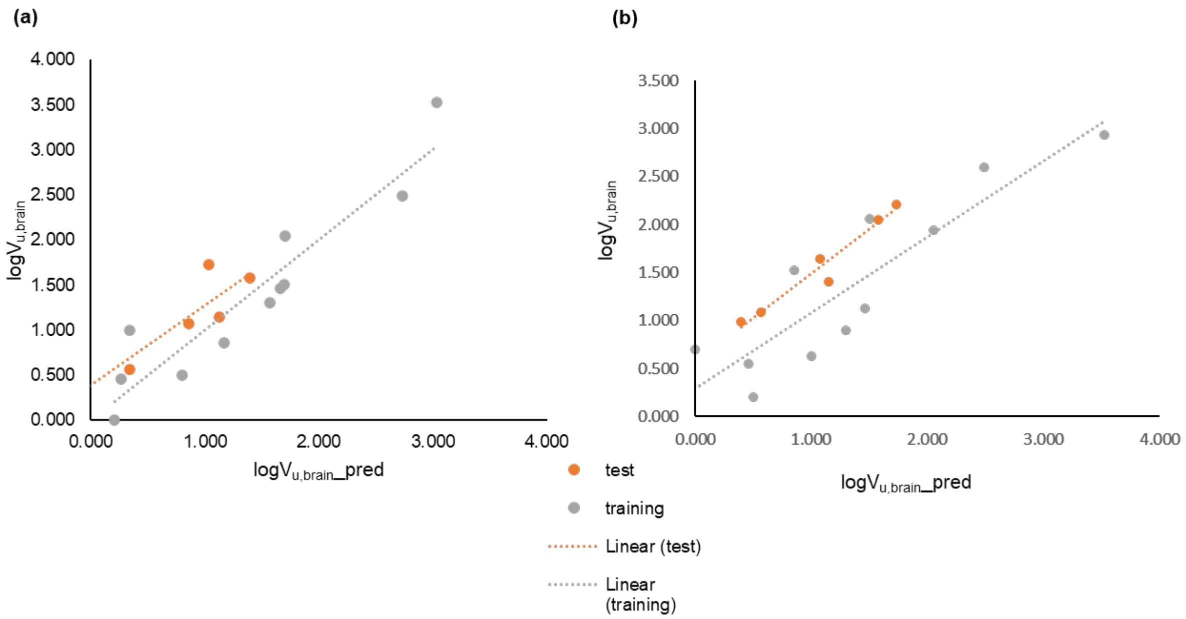

Figure S17. Representative plot of observed vs predicted logV$_{u,brain}$ values from external validation of (**a**) Eq. (15); (**b**) Eq.17.

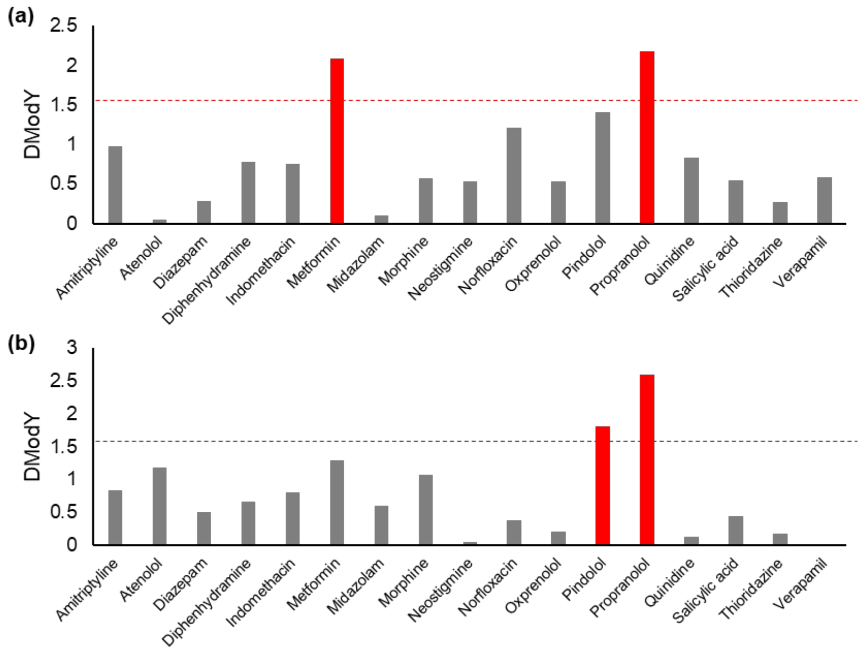

Figure S18. (a) DmodY bar plot for the PLS model 10 of logV$_{u,brain}$ prediction including IAM retention factors; (b) DmodY bar plot for the PLS model 11 of logV$_{u,brain}$ prediction including logP. The red dashed line indicates the critical limit and the compounds extending it are highlighted with red bars.

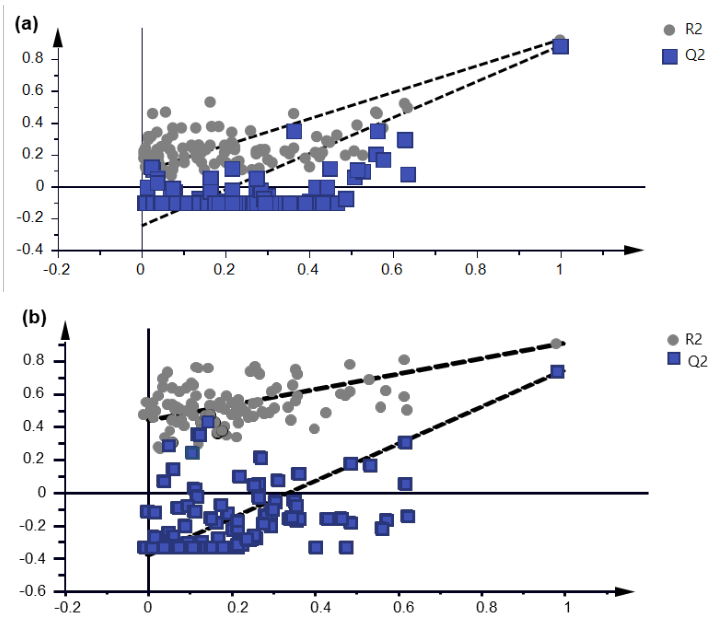

Figure S19. Permutation tests for the PLS models of logV$_{u,brain}$ prediction. (a) Permutation test (100 permutations) for the PLS model including IAM retention factors; **(b)** Permutation test (100 permutations) for the PLS model including logP.

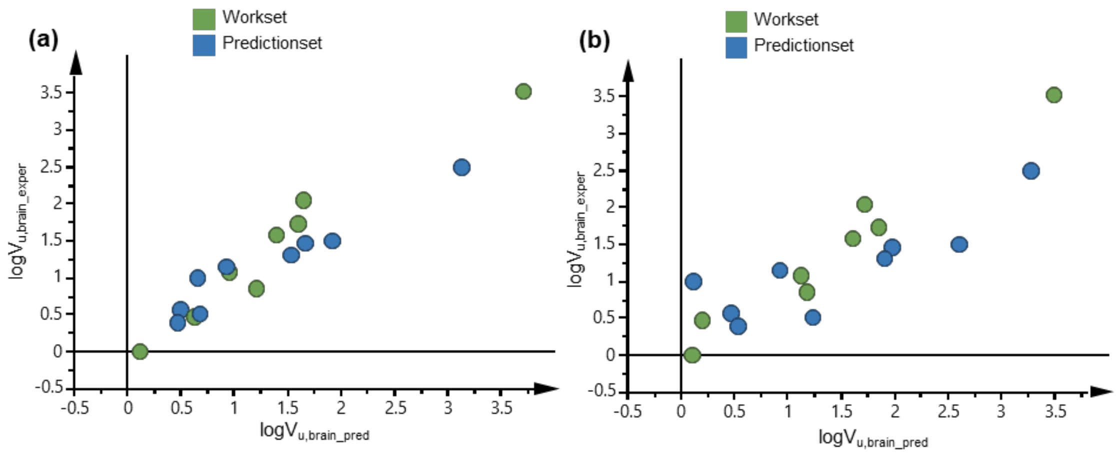

Figure S20. Representative plot of observed vs predicted logV$_{u,brain}$ values from external validation of PLS model including (a) IAM retention factors- PLS model 10; **(b)** lipophilicity parameters- PLS model 11.

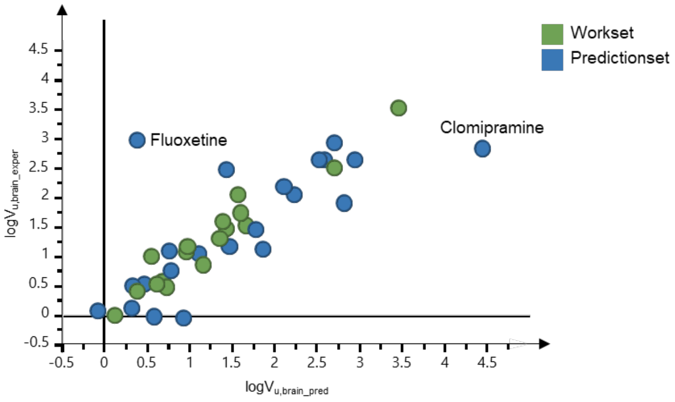

Figure S21. Plot of observed vs predicted logV$_{u,brain}$ values from PLS model 12. Experimental logV$_{u,brain}$ values were used as the training set and logV$_{u,brain}$ values predicted from Eq. (13) were used as the test set.

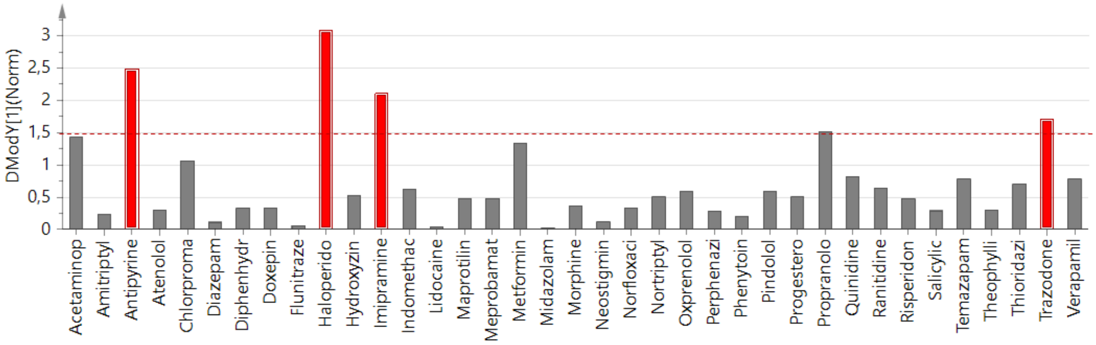

Figure S22. DmodY bar plot for the PLS model 12 of logV$_{u,brain}$.

Supplementary Tables

Table S1. Compounds included in the data set; pharmaceutical classification, chromatographic data and lipophilicity parameters.

Compound	Pharmaceutical class	logP	pH=7.4				pH=5.0	
			$logk_{wIAM}$	$logk_{10,HSA}$	$logk_{wAGP}$	logD	$logk_{wIAM}$	logD
Acetaminophen	Analgesic, antipyretic	0.51	0.18	-0.56	0.10	0.51	-0.06	0.51
Acetylsalicylic acid	Analgesic	0.90	-0.81	0.38		-1.20	-0.48	-0.29
Acyclovir	Guanosine analogue, antiviral	-1.56	-1.15	-1.33		-1.56	-1.37	-1.56
Aminopyrine	Analgesic	0.85	0.71	-0.30		0.63		0.86
Amitriptyline	Tricyclic antidepressant	4.92	2.99	0.89	2.40	2.50	2.06	1.13
Antipyrine	Antipyretic	0.56	0.45	-1.04	0.88	0.56		0.54
Atenolol	beta-1 selective blocker, treatment of hypertension	0.16	0.51	-1.04	0.10	-1.61	-0.20	-2.55
Atropine	Muscarinic antagonist	1.89	1.05	-0.36		-0.66		-1.76
Caffeine	Purine based stimulant	-0.07	0.26	-0.74		-0.07		-0.45
Candesartan	Angiotensin-receptor blocker, treatment of hypertension	3.36	1.00	0.42	0.09	-1.35	1.57	1.00
Chlorpromazine	Phenothiazine antipsychotic	5.41	3.33	1.34	2.30	3.38	2.58	1.84
Clomipramine	Tricyclic antidepressant	5.46	3.29	1.12	2.70	3.30	2.49	2.19
Cocaine	Ester local anesthetic	3.01	1.90	0.33		1.07		-0.96
Diazepam	Long-acting benzodiazepine with rapid onset	2.79	2.12	1.02	1.80	2.79	2.12	2.79
Diphenhydramine	H1 receptor antihistamine, treatment of seasonal allergies	3.4	1.82	0.14	1.87	1.29		-0.19
Doxepin	Tricyclic antidepressant and serotonin–norepinephrine reuptake inhibitor (SNRI)	3.96	2.50	0.56	2.30	2.22	1.98	0.86
Flunitrazepam	Benzodiazepine, anxiolytic	2.06	1.79	0.26	1.44	2.06		2.06
Fluoxetine	Selective serotonin reuptake inhibitor (SSRI) antidepressant	4.26	2.98	1.13	2.10	1.83	2.46	0.94

Haloperidol	Antipsychotic	4.28	2.65	0.77		2.98	1.86	1.15
Hydroxyzine	Antihistamine, anxiolytic	3.5	3.01	0.60		3.01	2.23	0.73
Ibuprofen	Nonsteroidal anti-inflammatory (NSAID) and non-selective COX inhibitor, analgesic	3.5	0.87	1.33		1.07	1.55	2.15
Imipramine	Tricyclic antidepressant	4.44	2.73	0.77	2.25	2.40	2.05	1.29
Indomethacin	Nonsteroidal anti-inflammatory (NSAID), symptomatic treatment of chronic musculoskeletal pain	4.27	2.41	1.76	1.59	1.03	2.93	2.95
Lidocaine	Local anesthetic	2.26	1.27	0.07	1.25	1.71	0.38	0.37
Lorazepam	Short-acting benzodiazepine, anxiolytic	2.51	2.03	0.67	1.33	2.51	2.01	2.51
Maprotiline	Tetracyclic antidepressant	4.14	2.81	0.80		1.44	2.25	1.19
Meprobamate	Anxiolytic	0.70	0.72	-0.36		0.70	0.54	1.07
Metformin	Biguanide antihyperglycemic (type 2 diabetes mellitus)	-0.91	-0.37	-1.37		-3.91		-3.91
Midazolam	Short-acting benzodiazepine, anxiolytic	3.27	2.77	0.99		3.27	1.93	1.95
Morphine	Opioid agonist, analgesic	0.89	0.63	-0.55		-0.06	0.10	-2.03
Naproxen	Nonsteroidal anti-inflammatory (NSAID)	3.18	0.98	1.55	0.20	0.33	1.71	2.24
Neostigmine	Cholinesterase inhibitor used in the symptomatic treatment of myasthenia gravis	-2.16	0.37	-1.91		-2.16	-0.08	-2.16
Norfloxacin	Broad-spectrum fluoroquinolone antibiotic	-1.03	1.01	-0.92		-1.03	1.31	-1.17
Nortriptyline	Tricyclic antidepressant	4.04	2.83	0.82	2.30	1.70	2.12	1.16
Ofloxacin	Antibacterial agent	-0.39	1.30	-0.65		-0.44	1.35	-1.31
Oxprenolol	Non-selective beta-adrenergic antagonist, treatment of hypertension, angina pectoris,	2.1	1.47	-0.25		0.32	0.87	-0.61

	arrhythmias, and anxiety							
Pentazocine	Analgesic	4.64	2.06	1.09		4.02	1.70	1.24
Pentobarbital	Barbiturate drug, sedative, antiepileptic	2.07	1.29	-0.04	1.35	1.95	1.40	1.88
Perphenazine	Phenothiazine antipsychotic	4.20	3.67	1.24	3.19	3.65		1.61
Phenobarbital	Long-lasting barbiturate and anticonvulsant	1.47	0.28	-0.22	1.10	1.14	0.71	1.47
Phenytoin	Anticonvulsant	2.24	1.75	0.44	1.60	2.17	1.94	1.95
Pindolol	Beta adrenoceptor antagonist, treatment of hypertension, edema, ventricular tachycardias, and atrial fibrillation.	1.75	1.47	-0.03		-0.21	0.73	-1.15
Piracetam	Nootropic cyclic GABA derivative	-1.54		-1.32		-1.54		-1.54
Progesterone	Endogenous steroid sex hormone	3.48	3.06	0.84		3.48		3.89
Promethazine	First-generation antihistamine	4.81	2.78	1.05	2.50	2.79	2.18	1.59
Propranolol	Non-selective beta-adrenergic antagonist	2.98	2.33	0.48	2.20	1.26	1.63	0.32
Quinidine	Class I antiarrhythmic agent	3.44	2.34	0.29	1.71	2.41		-1.30
Ranitidine	Histamine H2 antagonist	1.28	0.61	-0.41		-0.53		-3.37
Risperidone	Second-generation antipsychotic	2.50	2.49	0.13		1.63		-1.24
Salicylic acid	Salicylate, treatment of acne, psoriasis, calluses, corns, keratosis pilaris, and warts.	2.19	-0.08	0.34		-1.68	0.46	0.04
Scopolamine	Belladonna alkaloid with anticholinergic effect	0.55	1.15	-0.64		-0.23	0.73	-2.31
Temazapam	Short-acting benzodiazepine, anxiolytic	2.19	1.76	0.28		2.19	1.76	2.32
Theophylline	Xanthine analogue phosphodiesterase inhibiting drug used in therapy for respiratory diseases	-0.02	-0.08	-0.67		-0.02	-0.26	-0.02

Thioridazine	Phenothiazine antipsychotic	5.90	3.98	1.59		3.34		3.06
Trazodone	Serotonin uptake inhibitor, antidepressant	3.80	2.34	0.82	2.36	3.70		2.89
Verapamil	Non-dihydropyridine calcium channel blocker, treatment of angina, arrhythmia, and hypertension.	3.79	2.76	0.66	0.10	2.57	1.86	1.20

Table S2. Brain disposition data, i.e., $K_{p,brain}$, $K_{p,uu,brain}$, $f_{u,brain}$, $V_{u,brain}$ for the investigated compounds.

Compound	$K_{p,brain}$	$K_{p,uu,brain}$	$f_{u,brain}$	$V_{u,brain}$
Acetaminophen	0.49	0.18	0.832	
Acetylsalicylic acid	0.32			
Acyclovir	0.14	0.3		
Aminopyrine	1.00			
Amitriptyline	9.55	0.73	0.010	310.00
Antipyrine	0.80	0.71	0.862	
Atenolol	0.04	0.03	0.900	2.50
Atropine	0.87			
Caffeine	0.89	0.77		
Candesartan	0.04			
Chlorpromazine	11.48		0.003	
Clomipramine	21.88		0.004	
Cocaine	3.98	0.37		
Diazepam	3.31	1.02	0.042	20.00
Diphenhydramine			0.048	32.00
Doxepin			0.025	
Flunitrazepam			0.219	
Fluoxetine	5.25		0.003	
Haloperidol	21.88		0.008	
Hydroxyzine	2.45		0.013	
Ibuprofen		0.56		
Imipramine	6.76		0.035	
Indomethacin	0.05	0.11	0.042	14.00
Lidocaine	2.19		0.209	
Lorazepam	2.95			
Maprotiline	57.54		0.006	
Meprobamate			0.638	
Metformin			0.950	10.00
Midazolam	2.29	2.19	0.026	29.00
Morphine	0.69	0.32	0.760	3.70
Naproxen		0.60		
Neostigmine			0.950	3.20
Norfloxacin	0.07	0.05	0.580	2.90
Nortriptyline			0.006	
Ofloxacin		0.12		
Oxprenolol	1.06	0.2	0.280	11.80
Pentazocine	3.51			
Pentobarbital	1.32			
Perphenazine			0.004	
Phenobarbital	0.72			
Phenytoin	0.91	0.45	0.121	
Pindolol	0.72	0.5	0.400	7.20
Piracetam	1.00			
Progesterone			0.046	
Promethazine	6.67			

Propranolol	4.37	0.61	0.025	112.00
Quinidine	0.35	0.03	0.090	38.00
Ranitidine	0.06		0.955	
Risperidone	0.95	0.08	0.087	
Salicylic acid	0.08	0.19	0.630	1.00
Scopolamine	1.70			
Temazapam			0.054	
Theophylline	0.51	0.05	0.562	
Thioridazine	1.74	0.45	0.001	3333.00
Trazodone			0.071	
Verapamil	0.20	0.05	0.052	54.00

Table S3. Variable Importance to Projection (VIP) values in the PLS model 4 for $logK_{p,uu,brain}$ on the basis of biomimetic properties, lipophilicity and computational descriptors.

Variable	VIP	Variable	VIP
TPSA	1.398	WASA	1.29
SHBa	1.365	SHBint2	1.38
NOnr	1.35	Pos.Fract. 7.4	0.814
sumdelI	1.287	SASA	1.37
ka3	1.248	Nnr	1.41
sumI	1.227	gmin	0.967
phia	1.223	SAVol	1.33
Rotlbonds	1.161	A	1.29
DipoleY	1.16	SssO	1.6
O	1.158	PSASA	0.756
ka2	1.157	WAVol	1.32
ka1	1.134	hmax	1.26
SHCsats	1.133	Total Dipole	0.831
S	1.125	nvx	1.31
Onr	1.121	V	1.37
NO	1.121	molweight	1.29
SdssC	1.106	Ioniz Gr	1.44
logkAGP	1.1	SHother	0.961
DipoleX	1.079	SHBd	0.992
B	1.068	SHaaCH	0.95
logD5.0	1.043	logkHSA10ACN	0.886
HBA	1.036	HBD	1.03
logD7.4	1.01	Pos.Fract. 5	0.947
SwHBa	0.9934	SssCH2	1.3
SaaCH	0.9813	LogP	1.04
SHBint4	0.9762	$logk_{wIAM5.0}$	0.954
pKa	0.969	$logk_{wIAM}$	1.2

Table S4. Observed and predicted $\log K_{p,uu,brain}$ **values by the PLS model 4, based on computational descriptors.**

Compound	$\log Kp_{uubrain}$(obs)	$\log Kp_{uubrain}$(pred)
Acetaminophen	-0.745	-0,269
Acyclovir	-0.523	-0,868
Amitriptyline	-0.137	-0,0203
Antipyrine	-0.149	0,146
Atenolol	-1.495	-1,16
Caffeine	-0.113	-0,211
Cocaine	-0.432	-0,67
Diazepam	0.011	-0,261
Indomethacin	-0.959	-0,256
Midazolam	0.340	-0,863
Morphine	-0.495	-0,224
Naproxen	-0.222	-0,465
Norfloxacin	-1.27	-0,361
Ofloxacin	-0.921	-0,933
Oxprenolol	-0.699	-1,01
Phenytoin	-0.347	-0,835
Pindolol	-0.301	-0,343
Propranolol	-0.215	-0,467
Qunidine*	-1.523	-0.483
Risperidone	-1.097	-0,886
Salicylic acid	-0.721	-0,354
Theophylline*	-1.301	-0,185
Thioridazine	-0.347	-0,229
Verapamil	-1.276	-1,35

*Not included in the model

Table S5. Experimental and predicted logBB values by MLR and PLS models.

Compound	logBB exper.	logBB pred. Eq. (1)	logBB pred. Eq. (2)	logBB pred. PLS model 5	logBB pred. PLS model 6
**Acetaminophen**	-0.31	-0.15	0.00	-0.30	-0.27
**Acetylsalicylic acid**	-0.5	-0.57	-0.49	-0.88	-0.85
**Acyclovir**	-0.84	-1.53	-1.37	-1.09	-1.07
**Aminopyrine**	0.00	0.34	0.38	0.08	0.09
**Amitriptyline**	0.98	1.13	1.04	0.88	0.86
**Antipyrine**	-0.097	0.35	0.42	-0.07	-0.06
**Atenolol**	-1.42	-0.73	-0.89	-0.74	-0.81
**Atropine**	-0.06	-0.02	-0.18	0.32	0.22
**Caffeine**	-0.05	-0.30	-0.24	-0.12	-0.13
**Candesartan**	-1.4	-1.25	-1.40	-1.99	-1.99
**Chlorpromazine**	1.06	0.68	0.70	0.78	0.79
**Clomipramine**	1.34	1.12	1.10	1.00	1.01
**Cocaine**	0.6	0.02	-0.03	0.22	0.19
**Diazepam**	0.52	0.46	0.60	0.63	0.67
**Fluoxetine**	0.72	0.81	0.65	0.75	0.70
**Haloperidol**	1.34	0.41	0.50	0.79	0.82
**Hydroxyzine**	0.39	0.55	0.58	0.57	0.59
**Imipramine**	0.83	1.03	0.97	0.92	0.90
**Indomethacin**	-1.26	-0.13	-0.24	-0.80	-0.79
**Lidocaine**	0.34	0.33	0.45	-0.03	0.02
**Lorazepam**	0.47	-0.07	0.09	0.21	0.26
**Maprotiline**	1.76	0.94	0.74	1.08	0.99
**Midazolam**	0.36	0.62	0.72	0.46	0.51
**Morphine**	-0.16	-0.14	-0.15	-0.41	-0.41
**Norfloxacin**	-1.15	-0.44	-0.61	-1.18	-1.22
**Oxprenolol**	0.025	0.04	-0.05	0.10	0.05
**Pentazocine**	0.545	0.62	0.93	0.58	0.71
**Pentobarbital**	0.12	-0.43	-0.21	0.23	0.28
**Phenobarbital**	-0.14	-0.60	-0.33	-0.20	-0.13
**Phenytoin**	-0.04	-0.05	0.10	0.21	0.25
**Pindolol**	-0.14	-0.08	-0.24	0.01	-0.07
**Promethazine**	0.824	0.59	-0.54	0.81	0.82
**Propranolol**	0.64	0.34	0.62	0.65	0.59
**Quinidine**	-0.46	0.27	0.23	0.00	0.03
**Ranitidine**	-1.23	-1.24	0.34	-1.52	-1.52
**Risperidone**	-0.02	0.00	-1.21	0.07	0.05
**Salicylic acid**	-1.1	-0.34	-0.04	-0.73	-0.78
**Scopolamine**	0.23	-0.39	-0.46	0.11	0.04
**Theophylline**	-0.29	-0.55	-0.47	-0.19	-0.18
**Thioridazine**	0.24	0.34	-0.41	0.68	0.66
**Verapamil**	-0.7	0.01	0.29	-0.33	-0.28

Table S6. Experimental and predicted $\log K_b$ values by MLR and PLS models.

Compound	$\log K_b$ exper.	$\log K_b$ pred. Eq. (7)	$\log K_b$ pred. Eq. (11)	$\log K_b$ pred. PLS model 8	$\log K_b$ pred. PLS model 9
Acetaminophen	0.692	0.407	-0.006	0.505	0.218
Amitriptyline	-1.996	-2.148	-2.226	-2.089	-2.182
Antipyrine	0.792	0.522	-0.033	0.479	0.065
Atenolol	0.950	0.713	0.758	0.959	0.579
Chlorpromazine	-2.522	-2.468	-2.522	-2.292	-2.160
Clomipramine	-2.431	-2.326	-2.479	-2.284	-2.192
Diazepam	-1.359	-1.519	-1.247	-1.527	-1.334
Diphenhydramine	-1.298	-0.985	-1.365	-1.055	-1.491
Doxepin	-1.592	-1.587	-1.884	-1.636	-1.927
Flunitrazepam	-0.553	-0.586	-0.282	-0.645	-0.967
Fluoxetine	-2.584	-2.162	-1.684	-1.996	-2.108
Haloperidol	-2.083	-1.672	-2.071	-1.744	-2.034
Hydroxyzine	-1.881	-1.664	-1.779	-1.665	-1.886
Imipramine	-1.441	-1.828	-1.989	-1.792	-1.826
Indomethacin	-1.359	-1.858	-0.885	-1.727	-1.628
Lidocaine	-0.579	-0.534	-1.269	-0.447	-0.034
Maprotiline	-2.220	-2.000	-1.662	-2.035	-2.118
Meprobamate	0.245	0.338	0.458	0.528	1.098
Metformin	1.270	1.375	2.005	1.453	1.862
Midazolam	-1.574	-1.858	-1.559	-1.895	-1.822
Morphine	0.499	0.716	0.598	0.653	0.464
Neostigmine	1.270	1.154	0.864	1.138	0.992
Norfloxacin	0.139	0.486	-0.322	0.490	0.302
Nortriptyline	-2.227	-2.022	-1.803	-1.989	-2.085
Oxprenolol	-0.411	-0.356	-0.481	-0.283	-0.366
Perphenazine	-2.397	-2.375	-2.352	-2.448	-2.061
Phenytoin	-0.862	-0.887	-0.82	-0.997	-1.195
Pindolol	-0.177	-0.477	-0.193	-0.421	-0.340
Progesterone	-1.317	-2.047	-1.811	-1.630	-1.034
Propranolol	-1.592	-1.338	-1.182	-1.421	-0.966
Quinidine	-1.005	-1.128	-1.581	-0.917	-1.151
Ranitidine	1.317	0.538	0.596	0.672	0.640
Risperidone	-1.021	-0.898	-0.724	-0.751	-1.288
Salicylic acid	0.230	0.057	0.209	-0.122	-0.201
Temazapam	-1.244	-0.805	-0.731	-0.964	-1.037
Theophylline	0.107	0.946	0.85	1.043	0.710
Thioridazine	-3.174	-2.960	-2.501	-2.880	-2.615
Trazodone	-1.121	-1.193	-1.343	-1.164	-1.491
Verapamil	-1.261	-1.336	-1.318	-1.400	-1.157

Table S7. Observed vs predicted $f_{u,brain}$ correlation: $f_{u,brain} = a*f_{u,brain,pred} + b$.

	a	b	n	R^2	s
Eq. (7)- IAM retention	0.974(±0.039)	0.007(±0.017)	39	0.944	0.083
Eq. (11)- Lipophilicity	1.004(±0.060)	0.016(±0.024)	39	0.983	0.119
PLS- IAM retention	0.951(±0.040)	0.007(±0.018)	39	0.939	0.087
PLS- Lipophilicity	0.966(±0.057)	0.018(±0.024)	39	0.886	0.118

Table S8. Experimental and predicted $logV_{u,brain}$ values by MLR and PLS models.

Compound	$logV_{u,brain}$ exper.	$logV_{u,brain}$ pred. Eq. (15)	$logV_{u,brain}$ pred. Eq. (17)	$logV_{u,brain}$ pred. PLS model 10	$logV_{u,brain}$ pred. PLS model 11
**Amitriptyline**	2.491	2.607	2.383	2.745	2.701
**Atenolol**	0.398	0.206	0.792	0.443	0.695
**Diazepam**	1.301	1.637	0.822	1.415	1.173
**Diphenhydramine**	1.505	1.663	1.857	1.616	1.671
**Indomethacin**	1.146	1.391	1.32	1.152	0.946
**Metformin**	1.000	0.298	0.437	0.541	0.678
**Midazolam**	1.462	1.829	1.04	1.499	1.311
**Morphine**	0.568	0.494	0.908	0.689	0.838
**Neostigmine**	0.505	0.759	0.017	0.619	0.491
**Norfloxacin**	0.462	0.518	0.367	0.724	0.557
**Oxprenolol**	1.072	1.018	1.445	0.865	1.019
**Pindolol**	0.857	1.232	1.327	1.090	1.310
**Propranolol**	2.049	1.768	1.741	1.472	1.400
**Quinidine**	1.580	1.561	1.854	1.368	1.546
**Salicylic acid**	0.000	0.265	0.62	0.175	0.110
**Thioridazine**	3.523	3.011	2.72	3.470	3.481
**Verapamil**	1.732	1.396	2.003	1.608	1.727
